# Genes2Networks: connecting lists of gene symbols using mammalian protein interactions databases

**DOI:** 10.1186/1471-2105-8-372

**Published:** 2007-10-04

**Authors:** Seth I Berger, Jeremy M Posner, Avi Ma'ayan

**Affiliations:** 1 Department of Pharmacology and Systems Therapeutics, Mount Sinai School of Medicine, 1425 Madison Avenue, New York, 10029, New York, USA

## Abstract

**Background:**

In recent years, mammalian protein-protein interaction network databases have been developed. The interactions in these databases are either extracted manually from low-throughput experimental biomedical research literature, extracted automatically from literature using techniques such as natural language processing (NLP), generated experimentally using high-throughput methods such as yeast-2-hybrid screens, or interactions are predicted using an assortment of computational approaches. Genes or proteins identified as significantly changing in proteomic experiments, or identified as susceptibility disease genes in genomic studies, can be placed in the context of protein interaction networks in order to assign these genes and proteins to pathways and protein complexes.

**Results:**

Genes2Networks is a software system that integrates the content of ten mammalian interaction network datasets. Filtering techniques to prune low-confidence interactions were implemented. Genes2Networks is delivered as a web-based service using AJAX. The system can be used to extract relevant subnetworks created from "seed" lists of human Entrez gene symbols. The output includes a dynamic linkable three color web-based network map, with a statistical analysis report that identifies significant intermediate nodes used to connect the seed list.

**Conclusion:**

Genes2Networks is powerful web-based software that can help experimental biologists to interpret lists of genes and proteins such as those commonly produced through genomic and proteomic experiments, as well as lists of genes and proteins associated with disease processes. This system can be used to find relationships between genes and proteins from seed lists, and predict additional genes or proteins that may play key roles in common pathways or protein complexes.

## Background

The rapid increase in experimentally identified binary interactions between proteins has brought us to a stage where we are now able to start viewing how these interactions and components come together to form large functional regulatory networks [1]. However, it is impossible for researchers to keep up with the ever expanding literature. The emergence of high-throughput experimental technologies, such as yeast-2-hybrid screens [2,3], cDNA microarrays [4,5] and mass-spectrometry [6], as well as databases that mine legacy experimental literature [7,8] allow for the construction of large networks. Networks, formally graphs, are simple abstract representations of biomolecular interactions where cellular components are represented as nodes, and interactions connect these nodes through links.

The construction of cellular network datasets has several valuable uses. Network representation allows for extraction of global topological statistical and structural properties such as connectivity distribution [9], clustering [10], and the identification of network motifs [11] or graphlets [12]. These measurements provide clues about the design principles of intracellular organization. Interaction network datasets can also be used to predict unidentified interactions [13,14], or used as a starting point for quantitative computational modeling [15]. Additionally, interaction networks can assist in interpreting experimental results when identified lists of proteins or genes from proteomic or genomics experiments or computational studies can be placed in their contextual local interaction networks [16].

## Methods

**Figure 1:**
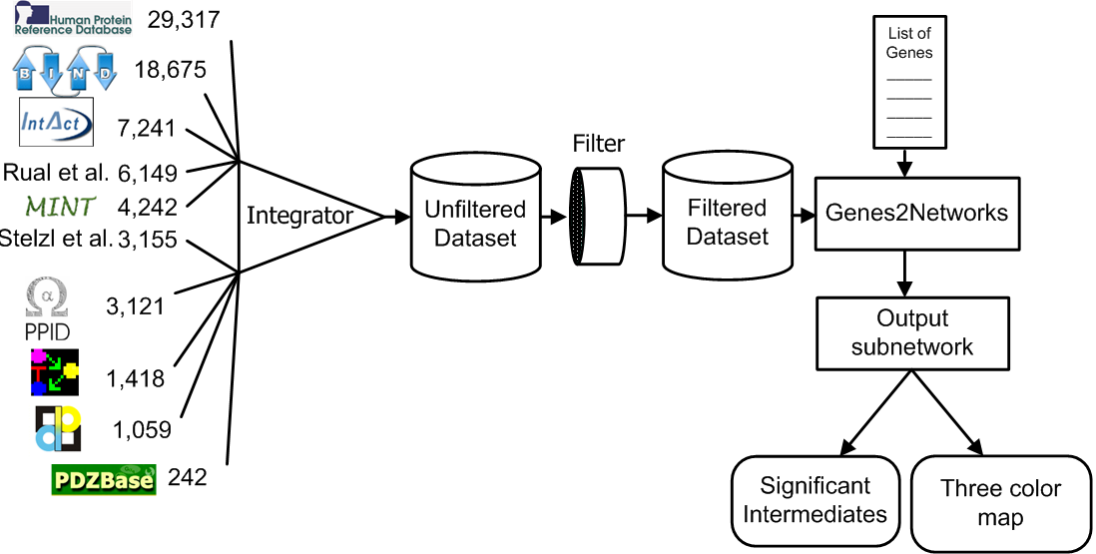
Ten mammalian PPI network datasets were consolidated into one dataset, and then filtered by excluding interactions originating from articles that contributed many interactions, or by excluding interactions with few references. The filtered merged dataset is then used to analyze lists of gene or protein names by outputting a subnetwork with nodes in three different colors: seed, significant, insignificant. The output also includes a statistical report that ranks intermediate nodes based on their specificity to interact with the seed list.

Our aim in developing the Genes2Network software is to provide cell- and molecular-experimental biologists as well as computational biologists with a user-friendly tool for creating subnetworks from lists of mammalian genes or proteins by connecting these genes or proteins using known protein-protein interactions. To accomplish this task we developed a large-scale high-quality mammalian protein-protein interaction database. This database was created by consolidating databases containing mostly low-throughput literature-based protein interaction data extracted manually by expert biologists, but also data generated from high-throughput methods. To develop Genes2Networks, we consolidated ten currently available mammalian protein interaction network datasets into one large dataset. To prune out interactions of low confidence, a simple filter was implemented. Genes2Networks is delivered as a web interface application. This tool can be used to extract relevant subnetworks given lists of gene or protein names. The input to the system is a list of Entrez gene symbols. The system uses the merged datasets made of selected databases to find interactions between the nodes in the seed list. The merged datasets can be filtered based on user preferences concerning the maximum number of interactions a reference can provide, and the minimum number of references required for interactions to be included. The resultant filtered dataset serves as a reference network for exploring, by depth-first traversal, paths between the seed nodes. Nodes that fall on paths shorter than a user defined path length between seed nodes are included as intermediates in the outputted subnetwork. The system's output includes a statistical analysis report, and a three color network map, highlighting the seed nodes in one color, the significant intermediates in another color, and the non-significant intermediates in a third color. The statistical analysis provides a list of intermediate nodes used to connect the gene names, sorted by significance of specificity to interact with nodes from the seed list. This process is illustrated in Figure 1.

### Developing a high-quality large-scale mammalian protein interaction network

We used only mammalian (mouse/rat/human) interactions recorded in the following datasets: BIND [17], HPRD [18], IntAct [19], DIP [20], MINT [21], Rual et al. [22], Stelzl et al. [23], Ma'ayan et al. [24], PDZBase [25], and PPID [19,26]. All interactions from these databases/datasets were determined experimentally and include a PubMed reference to the primary research article that describes the experiments used to identify the interactions. Some of the databases contain interactions that were manually extracted from the literature (e.g. HPRD); some datasets are the result of high throughput experimental data (e.g. Rual et al. and Stelzl et al.); whereas some databases contain both low and high-throughput interactions (e.g. BIND, IntAct, and DIP). Consolidation of interactions from the ten different network databases was accomplished by combining human/mouse/rat Entrez gene symbols using information from Swiss-Prot [27]. The consolidated network created from the ten datasets contains 44,877 interactions and 11,033 nodes. This network is stored in a structured text flat-file-space-delimited format. This file is loaded into the program using a hash data structure implemented in c language for fast loading and access. We do not include in this initial implementation datasets of interactions created via *in-silico ab-initio *interaction prediction methods or model organisms orthologs interactions such as those collected in OPHID [27], HPID [28], IntNetDB [29], and POINT [30]. The datasets we used describe mostly binary interactions, but in rare cases complexes containing more than two proteins are listed. These were excluded from the merged dataset. Nodes in the ten datasets are provided with accession codes linking them to entries describing genes and proteins in databases such as Swiss-Prot [31] and NCBI's Entrez Gene [32]. HPRD [18] and PPID [19,26] are not included in the public web interface application since these databases require a license for redistribution. Currently, HPRD and PPID data are only available to internal users at Mount Sinai School of Medicine.

### Filtering

Many of the interactions and components listed in the ten databases that we used are the result of high-throughput experiments such as yeast-2-hybrid screens [2,3], and mass-spectrometry [6]. These interactions are considered low-quality since these techniques often report many false positives [33]. Thus, we applied a simple filtering approach allowing users to exclude interactions originating from articles that provide many interactions, and/or include only interactions reported by several different papers. The rationale for this filtering approach is the assumption that a research article that reports many interactions is likely reporting the results of a high-throughput technique which tends to produce many false positives. Alternatively, interactions that are reported in many different research articles, and appear in multiple databases, can be given more confidence because these interactions have been reported multiple times independently. Hence, users may select to include only interactions from low-throughput studies with multiple references to improve the reliability of the consolidated network. Users are presented with list-boxes and text-boxes that allow adjustment of the filtering thresholds. More sophisticated filtering techniques implementing machine learning technologies such as support vector machines (SVM) [34], and taking into account more knowledge about the interactions (i.e. experimental method used, impact factor of journals, etc.) are planned for future implementations.

### Web interface

To enhance accessibility to the core Genes2Networks software, we developed a state-of-the-art web-based interface. This interface allows users to input lists of human Entrez Gene symbols in a textbox or through uploading a text file. As genes are added, the system validates the entries using NCBI's e-utils. The validation is achieved by searching the NCBI gene database, with the input entry, while restricting the organism to human. If an exact match is not found, the user is presented with a list of suggestions with links to choose the intended matching entry. By clicking on a highlighted gene symbol from the list of suggestions, the gene can be added to the seed list.

**Figure 2:**
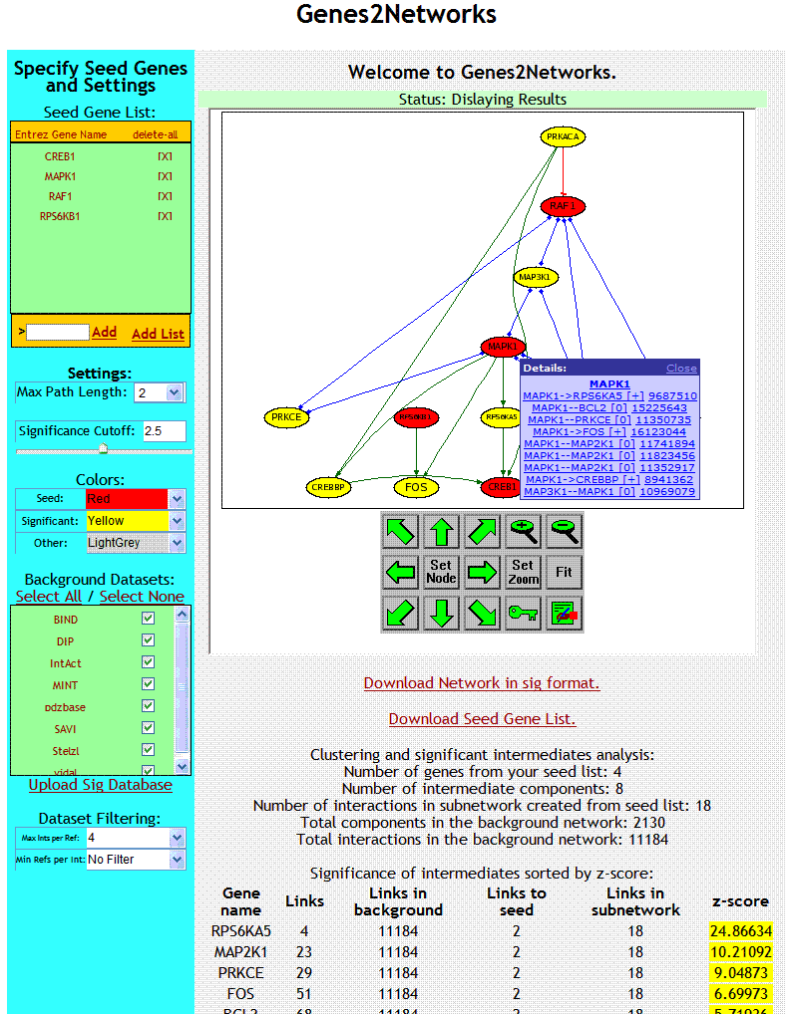
Genes2Networks web interface. The interface allows users to input a list of human Entrez Gene symbols, entered in a textbox or through a text file (top left). As genes are added, using the merged consolidated reference network made of different protein-protein interaction network databases, the program outputs a network map that visualize known interactions that "connect" the list of gene symbols from the seed list, and a statistical report that ranks intermediates based on their specificity to interact with the seed list.

Using the merged consolidated network reference database, the program outputs subnetworks that describe all found interactions and nodes on paths connecting the list of inputted gene symbols. The web interface provides users with full access to configure which databases to include in the consolidated reference network that is used to connect the genes. Additionally, users can upload other network databases for inclusion in the reference dataset. These additional networks can be consolidated with the provided networks. The output subnetwork is visualized using a dynamical web-enable AJAX viewer called AVIS [35]. The viewer allows browsing, zooming and panning, and linking to interaction resources. The user can configure the colors of the outputted nodes so that the seed-list genes, intermediate genes that are above a specified Z-score and the rest of the nodes are displayed in different colors. The user can also adjust the maximum number of steps/hops to use in order to find paths between the nodes in the seed list to connect the seed list genes. Steps/hops are the number links (not nodes) needed to connect the inputted seed list. Additionally, the program outputs a statistical report that ranks intermediates used to connect the genes based on their specificity to interact with the seed list. As the user adjusts the settings, changes in the resulting network are automatically redisplayed. A representative screenshot of the system is illustrated in Figure 2.

### Significant intermediates

The output subnetworks produced by Genes2Networks contain nodes, mostly proteins, which were not originally provided by the user as input. We call these nodes "intermediate nodes". Some of these intermediate nodes may be present in the output subnetwork because these intermediates are highly connected nodes (hubs) in the consolidated reference network used to connect the seed-list. On the other hand, intermediate nodes may be specific to interact with components from the inputted seed list. If these intermediates are specific, it may be beneficial for the user to identify them as potential specific regulators and specific participants in pathways, protein complexes and modules involving the input seed list. For this, Genes2Networks outputs a Z-score value of the significance of intermediates in the outputted subnetwork. The Z-score is computed for each intermediate node using a binomial proportions test [36] as follows:

z=(ac−bd)bd⋅(1−bd)d
 MathType@MTEF@5@5@+=feaafiart1ev1aaatCvAUfKttLearuWrP9MDH5MBPbIqV92AaeXatLxBI9gBaebbnrfifHhDYfgasaacH8akY=wiFfYdH8Gipec8Eeeu0xXdbba9frFj0=OqFfea0dXdd9vqai=hGuQ8kuc9pgc9s8qqaq=dirpe0xb9q8qiLsFr0=vr0=vr0dc8meaabaqaciaacaGaaeqabaqabeGadaaakeaacqWG6bGEcqGH9aqpdaWcaaqaamaabmaabaWaaSaaaeaacqWGHbqyaeaacqWGJbWyaaGaeyOeI0YaaSaaaeaacqWGIbGyaeaacqWGKbazaaaacaGLOaGaayzkaaaabaWaaOaaaeaadaWcaaqaamaalaaabaGaemOyaigabaGaemizaqgaaiabgwSixpaabmaabaGaeGymaeJaeyOeI0YaaSaaaeaacqWGIbGyaeaacqWGKbazaaaacaGLOaGaayzkaaaabaGaemizaqgaaaWcbeaaaaaaaa@4395@

Where "a" equals the links from the intermediate node being examined to nodes from the input seed list, "b" equals the total links for the intermediate node in the consolidated background reference network, "c" is the total links in the outputted subnetwork, and "d" is the total links in the consolidated background reference network. The outputted ranked list of intermediates is displayed underneath the subnetwork map viewer.

## Discussion and Conclusion

Several commercial and academic initiatives have been attempting to address the need for integration, consolidation, visualization, querying and organization of information about binary mammalian protein-protein interactions and signaling pathways from sparse sources. For example, Cytoscape [37] is Java-based desktop software for protein and gene network visualization. Cytoscape's several plug-ins allow for analysis and integration of experimental data as well as incorporation with Gene Ontology [38]. One Cytoscape plug-in, called cPath [39], is a data warehouse that joins together databases stored in PSI-MI XML format [19]. Other similar software platforms include: PIANA [40], Pathway Studio [7], ProViz [41], PATIKA [42], and Ingenuity. Some are commercial products and some were developed by academic laboratories and are freely available. Genes2Networks provides several advantages over existing systems; the consolidated network made from the ten databases, after filtering, is a high quality yet comprehensive dataset; the user interface is an intuitive web-based Web 2.0 enabled application; the systems is free for academic users; the system provides predictions about intermediate components and their involvement with the proteins and genes from seed lists by ranking intermediates according to their specificity to interact with the seed list. Genes2Networks is suitable for analysis of diverse proteomic and genomic experimental results. The web interface and visualization provide easy access and a user friendly environment eliminating the need for training.

## Availability and requirements

Project name: Genes2Networks

Project home page: 

Operating system: Platform independent

Programming language: C, JavaScript, PHP, Perl

Other requirements: The HPRD and PPID dataset are only available to Mount Sinai School of Medicine users due to licensing restrictions.

License: GNU GPL

Any restrictions to use by non-academics: License needed. Users should contact technology@mssm.edu

## Competing interests

The author(s) declares that there are no competing interests.

## Authors' contributions

AM designed and supervised the study, wrote the manuscript, implemented the significant intermediates statistical algorithm, and wrote the code for the initial Genes2Network prototype. JMP re-implemented and upgraded the code that merges and filters the datasets including implementing the hash function for fast loading of the datasets. JMP also rewrote the code to construct subnetworks from lists of gene names. SB designed and implemented the web interface including the AVIS visualization tool as well as upgraded the Genes2Network software to support listing of databases for interaction. SB also provided useful comments to the written manuscript.
